# Competition between influenza A virus subtypes through heterosubtypic immunity modulates re-infection and antibody dynamics in the mallard duck

**DOI:** 10.1371/journal.ppat.1006419

**Published:** 2017-06-22

**Authors:** Neus Latorre-Margalef, Justin D. Brown, Alinde Fojtik, Rebecca L. Poulson, Deborah Carter, Monique Franca, David E. Stallknecht

**Affiliations:** 1 Southeastern Cooperative Wildlife Disease Study, College of Veterinary Medicine, Department of Population Health, The University of Georgia, Athens, Georgia, United States of America; 2 Department of Biology, Lund University, Lund, Sweden; 3 Pennsylvania Game Commission, Pennsylvania State University, Animal Diagnostic Laboratory, University Park, Pennsylvania, United States of America; 4 Poultry Diagnostic and Research Center, College of Veterinary Medicine, Department of Population Health, The University of Georgia, Athens, Georgia, United States of America; St. Jude Children's Research Hospital, UNITED STATES

## Abstract

Our overall hypothesis is that host population immunity directed at multiple antigens will influence the prevalence, diversity and evolution of influenza A virus (IAV) in avian populations where the vast subtype diversity is maintained. To investigate how initial infection influences the outcome of later infections with homologous or heterologous IAV subtypes and how viruses interact through host immune responses, we carried out experimental infections in mallard ducks (*Anas platyrhynchos*). Mallards were pre-challenged with an H3N8 low-pathogenic IAV and were divided into six groups. At five weeks post H3N8 inoculation, each group was challenged with a different IAV subtype (H4N5, H10N7, H6N2, H12N5) or the same H3N8. Two additional pre-challenged groups were inoculated with the homologous H3N8 virus at weeks 11 and 15 after pre-challenge to evaluate the duration of protection. The results showed that mallards were still resistant to re-infection after 15 weeks. There was a significant reduction in shedding for all pre-challenged groups compared to controls and the outcome of the heterologous challenges varied according to hemagglutinin (HA) phylogenetic relatedness between the viruses used. There was a boost in the H3 antibody titer after re-infection with H4N5, which is consistent with original antigenic sin or antigenic seniority and suggest a putative strategy of virus evasion. These results imply competition between related subtypes that could regulate IAV subtype population dynamics in nature. Collectively, we provide new insights into within-host IAV complex interactions as drivers of IAV antigenic diversity that could allow the circulation of multiple subtypes in wild ducks.

## Author summary

Many features of pathogen diversification remain poorly explored although host immunity is recognized as a major driver of pathogen evolution. Influenza A viruses (IAVs) can infect many avian and mammalian hosts, but while few IAV subtypes circulate in human populations, subtype diversity is extensive in wild bird populations. How do these subtypes coexist in wild avian populations and do they compete within these natural host populations? Here we experimentally challenged mallard ducks with different IAVs to study how an initial infection with H3N8 determines the outcome of later infections (duration of infection and virus load) and antibody responses. There was complete protection to re-infection with the same H3N8 virus based on virus isolation. In addition, there was partial protection induced by H3N8 pre-challenge to other subtypes and development of heterosubtypic immunity indicated by significantly shorter infections and reduction in viral load compared to controls. This indicates that subtype dynamics in the host population are not independent. Amongst H3N8 pre-challenged groups, the highest protection was conferred to the H4N5 subtype which was most genetically related to H3N8. The H4N5 challenge also induced an increase in H3 antibody levels in that challenge group and evidence for original antigenic sin or antigenic seniority. Thus, previous infections with IAV can influence the outcome of subsequent challenge with different IAV subtypes. These results not only have relevance to understanding naturally occurring subtype diversity in wild avian populations but also in understanding potential outcomes associated with introduction of novel viruses such as highly pathogenic IAV H5 viruses in wild bird populations.

## Introduction

Diversification is a common feature in pathogen populations and this often involves the evolution of antigenic variants [1]. Examples of this exist in various pathogen systems: viruses (influenza A virus, Dengue virus, Bluetongue virus, and rotaviruses), bacteria (*Borrelia spp*, *Neisseria meningitis*, and *Pneumococcus*) and protozoan parasites (*Plasmodium spp*. and trypanosomes). Antigenic variation within specific hemagglutinin (HA) and neuraminidase (NA) subtypes is well described with influenza A viruses (IAVs) as this is an important consideration in developing vaccines and vaccination strategies associated with seasonal influenza viruses in humans and IAV affecting domestic livestock and poultry. Though multiple IAV subtypes circulate in these host systems, antigenic interactions between these subtypes are equally important but less understood. Shared epitopes between different HA subtypes associated with the HA stalk have been described and these may be important target epitopes for universal IAV vaccines. Immunity to these shared epitopes also may provide partial protection in subsequent infections with heterologous IAV [2] and could potentially influence clinical outcome and regulate subtype diversity in host populations through competition [3].

IAV have the capacity to infect many different hosts, from birds to mammals; however the vast majority of influenza subtype diversity is found in wild birds, especially waterfowl, gull, and shorebird populations where low-pathogenic IAV (LPIAV) representing 16 HA and 9 NA subtypes are maintained [4]. In these wild bird populations, many HA/NA subtype combinations co-circulate and it is notable that their abundance and relative diversity appears to vary over time and space [5–7]. In addition, individual birds are often co-infected or sequentially infected with multiple IAV subtypes in a given season or year [8, 9] and naturally infected mallards (*Anas platyrhynchos*) in the wild exhibit patterns of re-infections indicating heterosubtypic cross-immunity [9]. Experimental infections have demonstrated that initial infection with a specific IAV induces immune responses and protection against infection with homologous strains [10]. Additionally, initial viral infections could induce a partial protection to heterologous subtypes in experimental settings [10–13] and several studies have shown that LPIAV pre-exposure protects against a lethal highly pathogenic IAV (HPIAV) challenge [11, 14, 15]. Despite these observations little is known about the mechanisms, extent (strength and specificity) and persistence of these immune responses and the outcomes of re-exposures that have critical significance to understanding the maintenance of IAV antigenic diversity in multi-strain/subtype-pathogen systems such as occur with IAV and ducks.

In this study, mallards were pre-challenged with a strain of H3N8 LPIAV, one of the most common subtypes in waterfowl, and pre-challenged ducks were subsequently re-challenged with the same strain (homologous challenge) or with different strains representing various subtypes (heterologous challenges). Groups of ducks in the homologous challenge were exposed at different time intervals to evaluate long-term antibody responses and potential protection as well as to further investigate the potential influence of age [16]. The different strains used in the re-challenge were chosen to represented a gradient in the degree of phylogenetic relatedness between the HA and belonged to subtypes commonly found in waterfowl populations.

The objective of the present study was to experimentally mimic re-exposures that commonly occur in mallards and other ducks in nature to determine the effects of subsequent challenge on susceptibility, duration and intensity of viral shedding and to characterize the humoral immune response.

## Results

### Variation in virus load and duration of shedding

#### Homologous challenge

**Fig 1:**
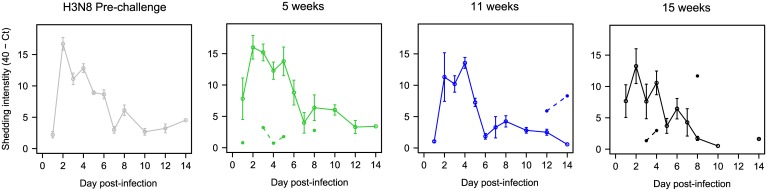
Pre-challenge reduces viral shedding in homologous challenge. Patterns of CL shedding in pre-challenged and control groups for different age and time intervals at re-challenge. The panels show the variation in intensity of shedding (40 –mean Ct-values per group and standard error of the mean (SE), no data points indicate negative RRT-PCR, lines are drawn between days with consecutive positive results) for the different groups: initial pre-challenge and 5-, 11- or 15- week intervals between H3N8 homologous infections. Continuous lines and circles indicate the control groups and dashed lines with filled dots indicate the H3N8 pre-challenged groups. This indicates that pre-challenge induced protection and suppressed CL shedding.

Mallards were initially inoculated at 4 weeks of age with LPIAV H3N8 (pre-challenge) and all were susceptible to infection and shed virus. The study design can be found as supporting information (S1 Table). Virus shedding was evaluated using an AIV Matrix Real-time Reverse Transcriptase PCR (RRT-PCR) protocol [17] and the PCR cycle threshold (Ct-value) was used as a proxy for virus load. Virus replication occurred in all the control groups for the H3N8 re-challenge where Ct-values were lowest in cloacal (CL) samples at 2 days post infection (dpi) (Fig 1), indicating a peak in virus load/shedding; as also seen in Fig 1, Ct-values thereafter increased over time showing that virus load/shedding decreased (Fig 1). None of the swabs from H3 pre-challenged groups were positive by virus isolation after the homologous re-challenge; however, some swabs tested positive by Matrix RRT-PCR (Fig 1; there were 12, 3, and 4 positive samples in the 5-, 11-, and 15-week re-challenge groups, respectively). In all cases (5-, 11-, and 15-week re-challenge), Ct-values were higher in pre-challenged birds than in controls indicating that pre-challenged birds shed fewer virus copies than naïve birds when challenged with the same strain.

The variation in Ct-values between groups was evaluated using Generalized Additive Mixed Models (GAMM) as described later in the methods section. The variables included in the models were: treatment group (pre-challenged or control), dpi and the interaction between both variables. A random effect was included for individual due to the repeated sampling of the same individuals over the course of infection. Model selection and comparison of the models were performed based on Akaike Information Criterion [18] corrected for small sample size (AICc). The best models with the lowest AICc score and tables with p-values are presented in S2–S4 Tables for all groups (5-, 11-, and 15- week re-challenges). Significant effects of group (p-value = 0.04) and dpi (p-value < 0.001) were observed for the 11- week group (S3 Table). Best models for the 5- and 15- week groups included effects of group (p-value = 0.001 and 0.06) and a significant interaction between dpi and group (p-value = 0.02 and 0.029) (S2 and S4 Tables).

In order to estimate the total shedding, the Area under the curve (AUC) was calculated (based on 40 minus the Ct-value). The AUCs were significantly lower for the pre-challenged groups for all three time intervals compared to controls (5- week interval 6.62 ± 0.71 compared to 101.19 ± 6.99 for controls, 11- week 6.38 ± 2.69 compared to 53.05 ± 7.1 for controls, 15- week 4.96 ± 3.19 compared to 45.25 ± 4.64 for controls) (Krustal-Wallis test [hereafter K-W], p-values < 0.001) (S1 Fig and S5 Table). In all groups, oropharyngeal (OP) shedding was generally lower than CL shedding (S2 Fig). Individuals did not show overt symptoms of disease and all gained weight throughout the trial.

**Fig 2:**
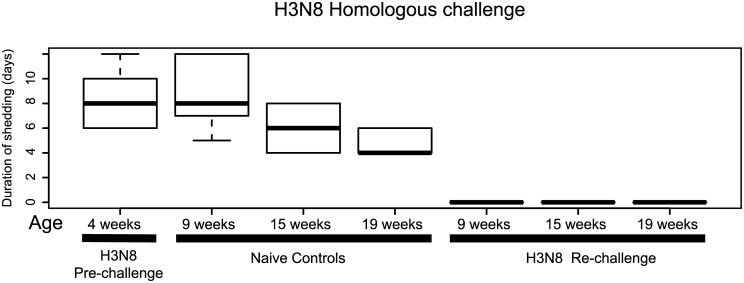
Duration of shedding for H3N8 homologous re-challenge groups and controls. Pre-exposure protects against homologous re-challenge in all groups based on virus isolation from CL samples. Duration of infection is shorter in older age groups. Box plots indicate the distribution of the values where the horizontal lines inside the boxes show the median value.

Based on comparisons of viral shedding of H3N8 during primary challenge (H3 pre-challenge and control groups), younger individuals (4 and 9 weeks of age) had significantly lower Ct-values (higher viral shedding) than older individuals (19 weeks) (Fig 1, S6 Table). Based on the best GAMM model by AICc, there were significant differences in Ct-values as a function of dpi and age group (S6 Table). Comparison of the AUC also showed significant differences in the total shedding between the age groups (mean and SE for the pre-challenge: 72.74 ± 5.22; 9 weeks of age: 101.19 ± 6.99; 15 weeks: 53.05 ± 7.1; 19 weeks: 45.25 ± 4.64) (K-W p-value < 0.001; S5 Table). The mean duration of infection for H3N8 in controls was 4.8 to 8.4 days (primary infections) (Fig 2, S5 Table) and there was a significant difference in the duration of infection between age groups (K-W p-value = 0.02) where the duration of infection was shorter for older birds.

#### Heterologous challenge: Subtype interaction

**Fig 3:**
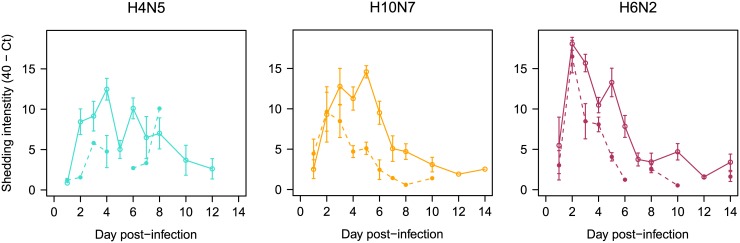
Pre-challenge reduces cloacal (CL) viral shedding after heterologous challenge. Birds were pre-challenged (dashed lines) or not (solid lines) with H3N8 and challenged with different LPIAV viruses/strains. CL shedding showing the variation in intensity of shedding: 40 –mean Ct-values and SE for the different heterologous challenge groups: H4N5, H10N7, H6N2. Continuous lines and circles indicate the control groups and discontinuous lines indicate the H3N8 pre-challenged groups.

All birds were susceptible to H3N8 infection in the pre-challenge and all were already negative by virus isolation on CL swabs on 21 and 35 dpi, prior to transfer to poultry isolators for the re-challenge trial. All viruses used in the re-challenge replicated in control birds as detected by virus isolation and RRT-PCR, except for the H12N5 strain (therefore data from the H12N5 groups could not be used for comparison, results for this group are shown in S2 Fig). For the H4N5, H10N7 and H6N2 viruses, Ct-values in H3 pre-challenged groups were high and higher than in the control groups indicating a reduction in virus replication and partial protection compared to controls (Fig 3). There were significant differences in Ct-values between the pre-challenged group H3N8 x H4N5 group and the H4N5 controls and best GAMM model included significant effects of dpi (p-value < 0.001), group (p-value = 0.022) and the interaction between dpi and group (p-value = 0.049), which means a difference in clearance rate for the different groups (S7 Table). For the H3N8 x H10N7 and H3N8 x H6N2 groups, the best models indicated significant differences in Ct-values when compared with their respective controls and both dpi (p-values < 0.001) and group (p-value = 0.001 and 0.024) had significant p-values (S8 and S9 Tables). Summaries of the model selection (AICc, ΔAICc, AICc weights) and significance p-values are provided as supplementary material (S7–S9 Tables). If differences in Ct-values from pre-challenged and controls are translated into log scale (as a difference of 3.3 Ct-values is equivalent to one log) the reduction in shedding ranges between 0.70 and 4.75 logs depending on the groups which corresponds to logs of Matrix RNA gene copies.

Further, the AUC estimates also showed significant differences for total shedding between pre-challenged and controls (mean and SE for the groups after challenge with: H4N5 8.63 ± 3.46 and 64.21 ± 9.3, H10N7 30.78 ± 7.62 and 66.57 ± 2.49, H6N2 36.76 ± 7.58 and 86.5 ± 2.58) (K-W p-values < 0.001) (S3 Fig and S5 Table) and support differences in shedding intensity with a lower shedding in pre-challenged groups.

**Fig 4:**
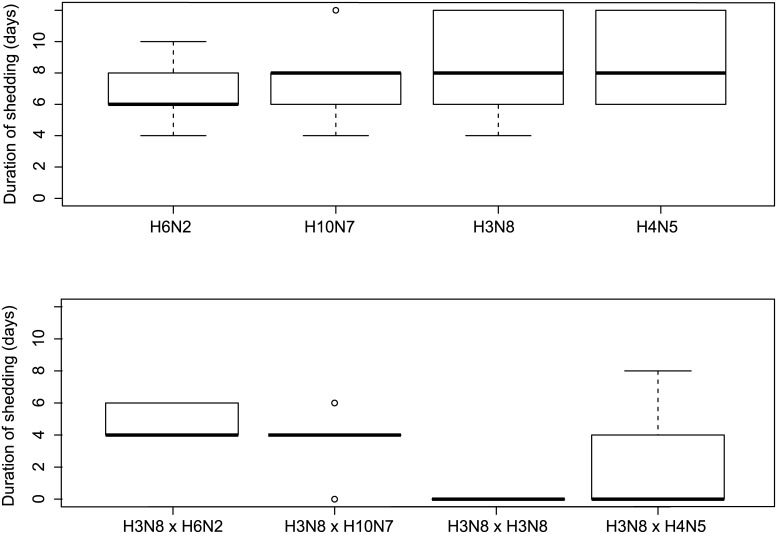
Pre-exposure reduces the duration of shedding. Duration of infection for viruses used in heterologous challenge (based on virus isolation for CL samples) in control groups (top panel) and H3N8 pre-challenged groups (lower panel). Box plots indicate the distribution of the values where the horizontal lines inside the boxes show the median value.

Mean duration of infection (as determined by detectable viral shedding, S5 Table) in the control groups at 9 weeks of age was 8 days (SE 0.66, range 7–8.8 days) while in the pre-challenged groups the mean duration of shedding was 2.6 days (SE 0.63) ranging from 0 days in the homologous challenge to 2.4 days (SE 1.6) for H3N8 x H4N5 group with only two individual birds infected (two CL samples), to 3.6 days for the H3N8 x H10N7 group (SE 0.98) where all individuals were infected (nine CL isolates recovered), and 5 days for H3N8 x H6N2 group (SE 0.58) where all individuals were also infected (nine CL isolates). There were no differences in the duration of shedding between the different subtypes (K-W p-value = 0.83) on the naïve control groups. There were significant differences in the duration of infection between pre-challenged groups (K-W p-value = 0.034) (Fig 4).

**Fig 5:**
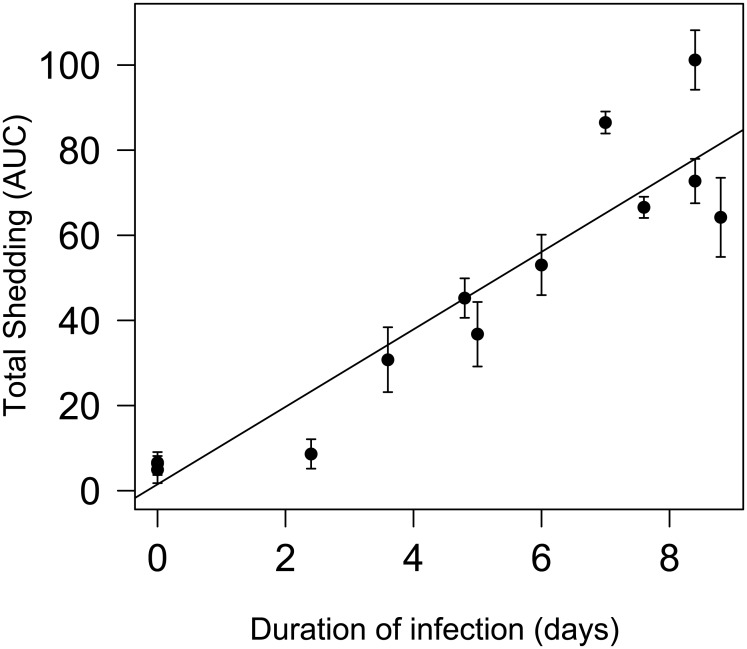
Correlation between total shedding and the duration of infection. The total shedding is expressed as the Area under the curve (AUC, mean value and SE) calculated for the different challenge groups.

Interestingly, total shedding (AUC) and duration of infection were significantly correlated (for both homo- and heterologous challenge groups) (Pearson’s correlation coefficient r = 0.92, df = 11, p-value < 0.001, regression n = 13, r^2^ = 0.86, p-value < 0.001) (Fig 5).

**Fig 6:**
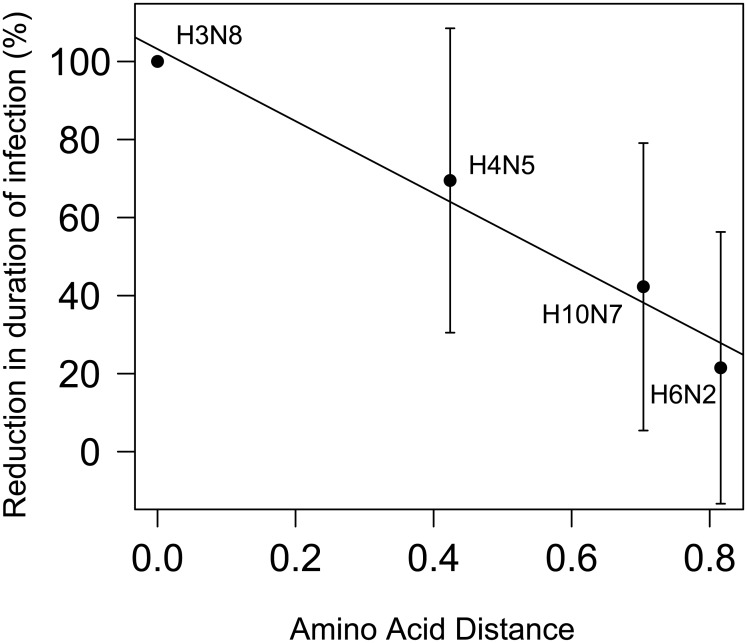
Correlation between amino acid distance between hemagglutinin (HA) subtypes in infections and the relative reduction in the duration of infection (mean and SE). This demonstrates that the reduction in duration of infection in the re-challenge is correlated to the similarity between HA involved in the challenges.

To test if genetic distance between HA subtypes was an important determinant in the strength of protection, the relationship between HA amino acid sequences and reduction of infection was evaluated. There was a negative correlation between the amino acid distance and the relative reduction in duration of infection per group ranging from 100% to 21.4% depending on the subtype in the re-challenge (Pearson’s correlation coefficient r = -0.98, df = 2, p-value = 0.014, regression n = 4, r^2^ = 0.97, p-value = 0.014),) (Fig 6).

#### Infectivity: Variation in Ct-values and virus isolation

We evaluated the factors that influenced isolation success of Matrix RRT-PCR positive samples using Generalized Linear Mixed Models (glmer function) including individual as random effect (S10 Table). There was a significant negative relationship between isolation and Ct-values (slope = -0.41 ± 0.005; Wald score [z] = -76.4, p-value < 0.001). As anticipated, samples with high Ct-values tended to have a lower isolation success that those with low Ct-values (high number of viral RNA copies). Remarkably, there were significant negative relationships in the model for both the H3N8 pre-challenge treatment (whether samples derived from primary or secondary infections) (intercept difference = -1.85 ± 0.005; Wald score [z] = -365.5, p-value < 0.001) and dpi (slope = -0.32 ± 0.005; Wald score [z] = -63.5, p-value < 0.001). This indicates that the probability of detecting IAV in RRT-PCR positive samples by virus isolation varies related to infection history and the days since infection. This was not dependent on sample type (CL or OP) (S10 Table).

### Serology

#### Long-term persistence of antibodies in the homologous challenge

**Fig 7:**
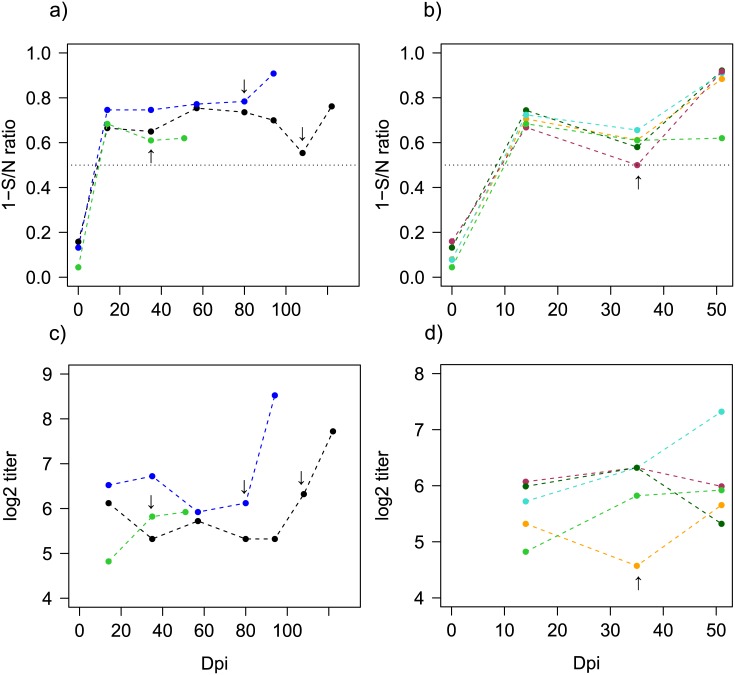
Antibody dynamics for the H3N8 homologous (panels a and c) and heterologous challenges (panels b and d). Data points indicate the average per group over time. Arrows indicate the timing of re-challenge for the different groups. The different colors indicate groups pre-challenged with H3N8 (green is the group re-challenged after a 5- weeks interval, blue are 11- weeks and black are at 15- weeks) or the different challenge groups: the groups challenged with H4N5 are light blue; H10N7 in orange; H6N2 in red; H12N5 in dark green and H3N8 (homologous challenge for reference) are green. Dashed lines are for H3N8 pre-challenged groups. a) and b) NP-ELISA ratios levels (expressed here as one minus the ratio) c) and d) H3 response by MN (log_2_ transformed titers) over time.

All individuals were seronegative to IAV prior to infection and seroconverted as measured by NP-ELISA on 14 dpi after pre-challenge and all but one also had H3 specific antibodies as measured by micro neutralization (MN) using the homologous antigen. NP-antibodies and H3 specific antibodies were detectable 5 weeks after initial H3N8 infection. There was no boost in the H3 antibody response in the 5- week interval group (5.82 ± 0.85 compared to 5.92 ± 0.5, paired t-test p-value = 0.326). For the 11- and 15- week challenge groups, there was a slight decrease in antibody titer over time with a boost and a significant increase in the H3 specific antibody response following re-challenge (tested differences in H3 MN titers before and after re-challenge: 11- week 6.12 ± 0.66 compared to 8.50 ± 0.58, paired t-test p-value = 0.003; 15- week 6.32 ± 0.31 compared to 7.70 ±0.36, paired t-test p-value = 0.032) (Fig 7, panels a and c and S11 Table with mean H3 log_2_ titer and SE).

#### Antibody responses to heterologous challenge

**Fig 8:**
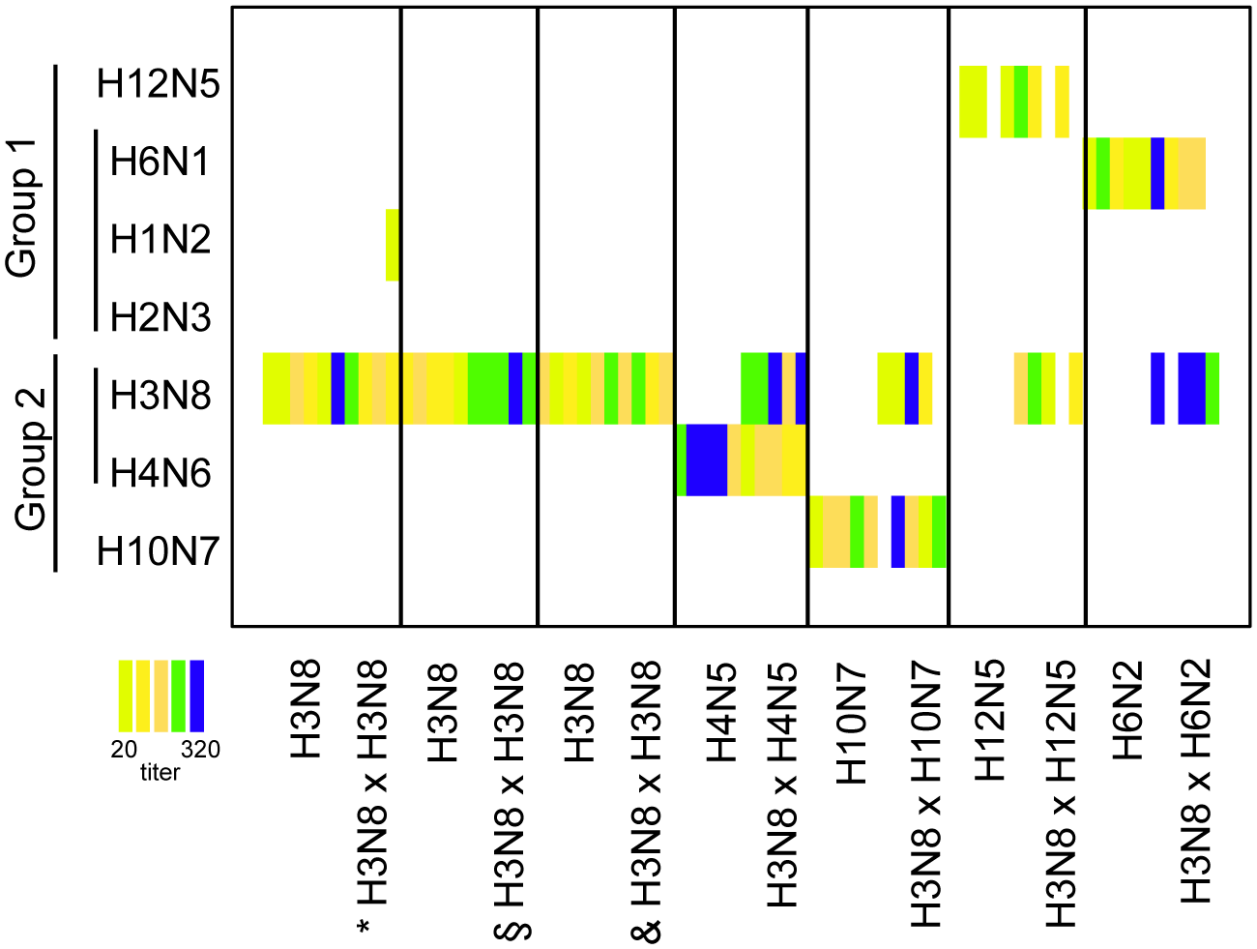
Heat map indicating virus neutralization profiles after secondary infection against a complete panel of H1-H12 and H14-H15 antigens for the different treatments (only antigens with positive MN included). Each box indicates a sample from an individual and the colors indicate the MN titers based on the legend. The challenge groups are indicated on the horizontal axis while the vertical axis indicates the antigens used in the neutralizations and their phylogenetic relationships. The homologous challenge 5- week interval is indicated by a *, 11- weeks by § and 15- week interval by &.

All individuals tested by IAV NP-ELISA were seronegative prior to infection. Individuals pre-challenged with H3N8 seroconverted on both NP-ELISA and H3 MN by 14 dpi. Based on the NP-ELISA response there was an increase in S/N ratios in all groups after the secondary challenge including the group challenged with H12N5 (Fig 7, panel b). There were no differences in H3 MN titers between groups of birds assigned to the different heterologous groups after initial H3N8 infection (K-W p-value = 0.261, 14 dpi) and prior to secondary infection (K-W p-value = 0.242, 35 dpi) (mean H3 titers and SE are presented S11 Table). An increase in the H3 MN titer was observed in the H3N8 x H4N5 challenge group because of an increase in the H3 MN titer for 3 individuals though there were no significant differences (6.32 ± 0.63 at 0 dpi and 7.32 ± 0.63 at 14 dpi, paired t-test p-value = 0.200) (Fig 7, S11 Table); antibodies to H4 were also detected (Fig 8). Similarly the group H3N8 x H10N7 did not show a significant increase in the H3 MN titer (4.57 ± 0.22 at 0 dpi and 5.66 ± 0.51 at 14 dpi, paired t-test p-value = 0.997). To evaluate the variation in H3 MN titers between H3 pre-challenged groups and the influence of time (i.e. prior to and after re-challenge) we used as before GAMM models. The best model included a significant effect of dpi (p-value = 0.02, S12 Table). All groups and most individuals developed detectable antibodies against the challenge subtype used in secondary challenges (Fig 8). MN results from sera collected at the termination of the experiment using a panel of H1-H15 prototype strains are shown in Fig 8. Most individuals had antibodies specific to the HA subtypes they were challenged with; however, one sample in the homologous group (5 week interval) was detected MN positive to H1N1.

## Discussion

### Homologous challenge

Here, we studied the persistence of protection to homologous IAV challenge by initially infecting mallards with H3N8 and re-challenging three groups with the same virus at different time intervals. The initial H3N8 induced long-term protection against homologous re-infection for up to 15 weeks post-challenge, which is much longer than expected. None of the re-challenged birds, regardless of time interval, shed virus as determined by virus isolation and that was true for all age groups and time intervals. Although some RRT-PCR positives were detected in re-challenged individuals, the Ct-values were high and significantly higher than in control groups indicating a low number of RNA copies possibly associated with non-infective virus. The observed protection and detectable antibody response is inconsistent with results from previous studies that concluded that infection conferred no protection against re-infection and that detectable antibody responses in ducks were short-lived [19, 20]. It was considered that antibody responses may not be detectable or protective due to the truncated structure of some of the IgY forms. A possible explanation of this inconsistency is that these truncated antibodies neutralize IAV but lack Hemagglutinin Inhibitory (HI) activity and the proportion between these forms vary over time being the truncated from most prevalent later in the immune response [21]. Our results are in agreement with other studies in both ducks and gulls that reported partial to complete protection against re-infection depending on the viruses, time between infections, host species and age and detection method [10, 22, 23]. The long-term persistence of antibodies after natural infection, artificial challenge or vaccination has been reported in captive birds up to 6 to 9 months post-exposure [24–26] though it is not know which parameters (i.e. HI or MN titers) are correlated to protection. When examining patterns in H3N8 infections for the different age classes in the naïve controls, all individuals were susceptible and competent to infection. The duration of infection and viral load in controls varied according to age and birds of 5 and 9 weeks of age experienced the highest replication based on AUC and duration of infection. This is consistent with earlier findings and indicative of older birds being more resistant to infection and having shorter infections [16]. This age effect could influence subsequent transmission risk and subtype diversity in cases were subtypes display seasonal patterns.

### Heterologous challenge

Results from IAV heterologous challenge experiments of ducks, geese and gulls have reported varying levels of partial protection upon re-challenge (11, 29, 30, 35). Such protection has also been reported from field-based studies [9, 27] and from experimental work where birds were challenged with HPIAV and effects could be measured by morbidity/mortality responses[11, 14]. It has also been reported that the order in which IAV subtypes are used to challenge the host is important. For instance, H3 appears to be poorly immunogenic and less protective as a primary infection compared to H4 that induced a stronger protective response [13]. The variation in reported protection from previous studies may be the result of asymmetric responses associated with strain or subtype specific variation that may be dependent on the virus causing primary infections [12, 13, 28].

We observed a partial protection to re-challenge in the individuals subsequently infected with heterologous subtypes indicating development of heterosubtypic cross-immunity by H3N8. When assessing the outcome of re-challenge there was a significantly lower viral load and shorter duration of infection in CL samples for all the groups pre-challenged with H3N8 compared to the naïve controls. This shorter period of detectable viral shedding is in agreement with estimates from the field [29, 30] and was observed for all re-challenged viruses.

The extent of partial protection, as measured by a decrease in duration of detectable viral shedding following re-infection was influenced by the genetic relatedness of the HA. This suggests immune mediated competition through cross-reactive responses [31, 32] and this could be related to both acquired humoral and cell mediated mechanisms [33]. Broadly neutralizing antibodies have been defined across HA groups [34] and within HA group [35, 36] that target conserved epitopes in the stalk [37, 38]. HA stalk antibodies are boosted upon re-infection and it is therefore thought that cross-reactive anti-stalk as well as anti-NA antibodies [39] can diminish the severity of disease in re-infections. Studies in humans have found consistent patterns of cross-immunity within HA group with increased severity of the disease in cohorts exposed with an HA of the opposite group during childhood when studying age-specific mortality caused by 1918 H1N1 and by HPIAV H5 and H7 [40, 41]. It has been proposed that extinction of influenza strains in humans could be driven by population immunity by HA stalk and NA antibodies [39] through competitive exclusion between strains [3]. Moreover, initial infections with a specific virus increases the probability of later infections by viruses from a different HA clade and group in wild mallards [9]. Comparable processes are likely acting in the avian IAV system where lineage replacement with strains from different continents has been reported [42, 43]. Theory predicts that antigenic variants tend to organize as discrete non-overlapping strains [1] in populations where cross-reactivity between viruses induces competition (i.e. case of H13 and H16) contrasting to the situation where cross-reactivity induces enhancement or facilitation, like Dengue, and variants may be antigenically clustered [44]. Here, after the heterologous re-challenge mallards shed viruses even if they were able to clear infections rapidly. An implication of this is the potential for selective processes like viral escape and antigenic drift to act in the same way as leaky vaccines [45, 46] which in turn could drive antigenic evolution as observed for H5N1 HPIAV [47] or similarly for seasonal H3N2 in humans [48]. Indeed, the estimates of divergence for some HA subtype indicate that divergence is relatively recent [40].

### Infectivity

Isolation success from RRT-PCR positive samples from secondary infections (re-challenge), as expected, was correlated with Ct-value, but was significantly lower compared to isolation results from primary infections in controls. This discrepancy has implications when interpreting field data and assigning infection states based on molecular diagnostics rather than virus isolation as uncertainty needs to be incorporated [49] and may explain why isolation rates from PCR positive samples often vary. This relationship also corroborates findings from the field where samples from adults have a lower isolation success than samples from juveniles [50] and where RRT-PCR positive samples used in experimental trials have not infected ducks [51]. It is therefore prudent to be cautious when using transformations of Ct-values (proxy for RNA copies) to EID_50_/ml or TCID_50_/ml by a standard curve based on a virus grown at optimal conditions such as cell culture or embryonated eggs as infectivity in hosts varies according to several parameters (age of host, previous exposure, specific virus and infection dose…).

### Antibody dynamics

Antibody levels (NP and H3 MN) after H3N8 infection decreased over time but most individuals remained positive for the duration of the experiment (15 weeks); all birds remained protected against homologous challenge. There was a significant boost in the antibody responses after homologous challenge for the long-term groups (11 and 15 weeks) but not for the group re-infected after 5 weeks where a rapid blocking of the infection may not have activated antibody recall. Additionally, the hyperimmune sera after homologous challenge did not cross-react with other IAV subtypes, except for one individual positive by H1 at a low titer of 20.

The heterologous re-infections resulted in a boost in the NP-antibody responses in all H3 pre-challenged groups. Interestingly most of the individuals had neutralizing antibodies against the HA antigens they had been exposed to. This also includes the H12N5 that replicated poorly but resulted in serological imprinting in three of the five individuals per group. Additionally no cross-reactivity to other subtypes was observed when tested by MN with a panel of HA (H1-H12 and H14-H15). H3-specific antibodies were detected by MN after H3N8 primary infection and persisted in the majority of individuals until the end of the experiment. There were interesting H3 patterns of antibody dynamics for the H3N8 X H4N5 group that showed a boost in H3 titer (Fig 7) consistent with original antigenic sin (OAS); H4 antibodies were also detected in this challenge group (Fig 8). This phenomenon of interference was first described after sequential influenza re-infections in humans [52, 53]. Older individuals can have a broader immunity through repeated exposure with highest titers to the strains individuals were exposed to early in life. These “senior strains”, are a singularity known as antigenic seniority or imprinting [40, 54]. Currently, we report OAS in avian hosts and between different HA subtypes (H3/H4 and possibly for H3/H10), however we expect that phenomenon could also arise between other IAV subtypes. Thus the order in which individuals are challenged with a specific virus could influence the future recognition of viruses and the specificity of the responses that ultimately shapes the outcome of later exposures in life. Since population immunity influences the emergence and spread of new strains and can influence vaccination success, it is critical to have a better understanding of these processes in different host species. We need to increase our understanding of cross-reactivity patterns and boost dynamics in re-infections to ultimately predict risks of IAV spread into different host populations in a context of non-naïve populations, for instance by using antibody landscapes [55].

The high degree of heterosubtypic immunity and subsequent competition found between common HA subtypes from ducks indicates that the transmission success and perpetuation of any subtype is dependent on the other viruses in the population and may explain the cyclic or chaotic nature in the prevalence of some subtypes. Partial immunity or complete immunity induced by pre-infection may reduce transmission potential in subsequent infections but also may promote the high degree of IAV antigenic diversity observed in wild avian populations. This competition may also result in subtype succession over time, like the predominance of H3 and H4 in fall migration [5–7] and spring blooming of other subtypes within Group 2 such as H7 or H10 [56]. With equal strength of HA immunity to all subtypes the present antigenic diversity found in wild birds would be unlikely. Surprisingly, the results from H13 and H16 experimental infections in black-headed gulls showed limited cross-immunity between subtypes and suggest independent cycles for these viruses [22].

However, for some strains or subtypes, cross-immunity may not be the only factor explaining their dynamics in the pathogen population. Pathogen fitness or success is usually measured by transmission risk that could be based on different transmission parameters such as the duration of infection and pathogen load [57] as well as how long viruses could remain infectious in the environment [58, 59]. We believe that virus fitness linked to host-specificity and functionality of IAV proteins [60] is also playing an important role as evidenced by the fact that the H12N5 IAV used in this study did not successfully replicate in mallards even though it was isolated from that host.

A possible interpretation of these results is that population immunity can reduce the probability of transmission and potential introduction success of exotic antigenic variants [61]. Previous studies have reported protection to HPIAV induced by pre-exposure to LPIAV strains in different bird species [11, 14]. In the context of H5N8 HPIAV clade 2.3.4.4 or other HPIAV the present results suggest that cross-immunity could also reduce viral shedding and contribute to stopping the spread of specific virus in wild ducks that have naturally been exposed to LPIAV [62–64].

We believe that the competitive processes described here and in other studies occur in nature; however, in natural host populations the complexity of the system increases due to the extensive subtype diversity of co-circulating viruses that these birds are continuously exposed to. Based on our results we propose that the extent of competition at individual level through host immunity could be determined by many different interacting parameters: the strains involved in infections, the exposure history (or boost responses like OAS) and likely time between exposure/s (assuming that immune memory decreases over time). These are many of the same factors that are considered in evaluating immune responses and protection against influenza in humans and domestic animals.

## Material and methods

### Virus selection, stock preparation and titrations

All LPIAV viruses used in these trials represented common North American subtypes and were originally isolated from wild mallards in Minnesota, USA [6]. Viruses included: A/Mallard/MN/AI07-4724/2007 (H3N8), A/Mallard/MN/AI11-4213/2011(H4N5), A/Mallard/MN/AI11-4982/2011(H6N2), A/Mallard/MN/AI11-4412/2011(H10N7) and A/Mallard/MN/AI11-3866/2011(H12N5). Virus stocks were propagated by a second passage in specific pathogen free (SPF) embryonated chicken eggs (ECE). Stocks were endpoint titrated using the Reed and Muench Method [65] in ECE to determine the median egg infectious dose (EID_50_/0.1 ml).

Ducks were inoculated with 0.1ml at an approximate dose/dilution of 10^6.0^ EID_50_/0.1ml; the inoculum was split between intranasal and oropharyngeal routes. Previous work has shown that different routes of infection (intranasal, intratracheal, intraocular, intracloacal, or intra-ingluvial) result in 100% infection and similar shedding patterns [66]. Based on back titration, the titers of the inocula, expressed in EID_50_/ 0.1 ml, were 10^5.8^ for H3N8 pre-challenge, 10^6.5^ for H12N8 and H6N2, 10^6.4^ for H3N8 (5 week challenge), 10^6.8^ for H10N7, 10^6.0^ for H3N8 (11 week challenge), 10^6.2^ for H4N5 and 10^6.3^ for H3N8 (15 week challenge).

The HA and NA segments of the isolates were amplified [67] and later sequenced using Sanger method. Geneious (version mac6_4_8_0_4) was used for sequence alignments and to calculate amino acid distances. Sequences are publicly available in GenBank (KX814369-KX814375).

### Ethics statement

All procedures were in accordance with the Animal Welfare Act and US regulations. All protocols for raising, infecting, and sampling mallards were approved by the Institutional Animal Care and Use Committee at the University of Georgia (UGA; AUP#: A2013 05-021-Y1-A1).

### Experimental infections

One-day-old mallards (Murray McMurray Hatchery, Webster City, IA, USA) were raised in captivity at the animal resources facilities of the College of Veterinary Medicine, UGA. Food and water were supplied ad libitum. All individuals were identified with bands and unique ID numbers. Individuals did not exhibit behavioral changes or overt symptoms of disease during the duration of the trial. All birds gained weight before and throughout the challenge studies. At four weeks of age, 40 ducks were moved to Biosafety Level (BSL) 2+ facilities and were pre-challenged with the H3N8 LPIAV.

The study design included re-challenges with homologous and heterologous viruses (S1 Table). Prior to the re-challenges, ducks were randomly divided into groups (five individuals per group, approximate ratio 1:1 females: males) and were moved into BSL 2+ poultry isolators at the Poultry Diagnostic Research Center, Athens, GA, USA that are intended to house up to five adult size mallards. After 2 days of acclimation, the re-challenge was conducted as previously described [12]. Each pre-challenged group had a control group (i.e. age matched naïve individuals). Ducks were humanely euthanized at the end of all challenge trials at 14 dpi following protocols approved at the UGA. For the homologous challenge, one group of five ducks was challenged with the same H3N8 at five weeks after the initial H3N8 pre-challenge and two additional groups were re-challenged with the H3N8 virus at weeks 11 and 15 post-H3N8 inoculation. Groups in the heterologous challenges were inoculated five weeks after initial H3N8 infection with subtypes representing different levels of relatedness between the key antigenic proteins HA and NA of the H3N8 used in the pre-challenge: H4N5, H10N7, H6N2 and H12N5.

### Sampling, extraction and virus isolations

Swabs were collected from the cloaca (CL) and oropharynx, (OP) and were placed in separate tubes containing 2 ml BHI transport media supplemented with antimicrobials [12]. Swab samples were collected on 0–8, 10, 12 and 14 and 21 (only after H3N8 pre-challenge) days post infection (dpi) and kept cold until transfer to the laboratory where they were stored at -80 C° until processing.

Virus isolations was performed on swabs from all birds during all challenges on 0, 2, 4, 6, 8, 10, 12, 14 dpi as previously described [68] using two 9 to 11 days-old SPF ECE. To insure that all birds were IAV negative before any bird movement or subsequent challenge, all birds were tested by virus isolation on 21 dpi after the H3N8 pre-challenge and on 0 dpi prior to all subsequent viral challenges.

### RNA extraction and RRT-PCR

Viral RNA from samples collected on 1 to 14 dpi were extracted using the MagMAX-96 AI/ND Viral RNA Isolation kit (Ambion, Austin, TX, USA) on the Thermo Electron KingFisher magnetic particle processor (Thermo Electron Corporation, Waltham, MA, USA) [68]. Negative (BHI media) and positive (diluted isolate) controls were included in each extraction. Molecular detection of the IAV Matrix gene by Real-time Reverse Transcriptase PCR (RRT-PCR) [17] was conducted with the Qiagen OneStep RT-PCR kit (Qiagen, Valencia, CA, USA) and the Cepheid SmartCycler System (Cepheid, Sunnyvale, CA, USA) [68]. Negative and positive controls were used on the extraction step and an IAV Matrix gene transcript (National Veterinary Services Laboratory, Ames, IA, USA) was included as positive control in the RRT-PCR. Ct–value stands for the cycle threshold when there is initial detection of the fluorescence signal at the beginning of the exponential phase of DNA duplication and is proportional to the initial number of RNA copies in a sample. Ct-values were therefore used as a proxy for viral load (S4 Fig). By using the same detection protocol and stock of the Matrix gene transcript (NVSL, Ames, IA, USA) it was previously established that Ct-values significantly correlate with the number of Matrix RNA gene copies when diluting the Matrix gene RNA transcript to generate a standard curve [57]. Samples with cycle (Ct) values lower than 40 were considered positive

### Serology

Serum samples were collected from the right jugular vein and transferred into serum separator tubes (Becton, Dickinson and Company, Franklin Lakes, NJ, USA), centrifuged upon arrival to the lab and serum was stored at -20 C° until analysis. Serum samples were taken immediately prior to H3N8 pre-challenge and prior to all subsequent challenges as well as at 14 dpi and between challenges for the homosubtypic long-term groups. Serum samples were tested with a commercially available nucleoprotein (NP)-ELISA kit (bELISA; FlockChek AI MultiS-Screen antibody test kit; IDEXX Laboratories, Westbrook, ME, USA). Specific antibodies against the different HA subtypes used in the trial were detected using a virus microneutralization (MN) assay in Madin Darby Canine Kidney cells (MDCK; ATCC, Manassas, VA, USA) as described previously [61]. Sera were additionally tested using the same viruses used for inoculation in the challenge and with prototype strains for H1-H12 and H14-H15 [69] (S1 Appendix) to detect cross-reactivity. These antigens also were prepared in MDCK and tests were run using an antigen concentration of 100 TCID_50_ /25 μl.

### Statistical analysis

All analyses were run on the R software [70] using the GAMM, nlme and lme4 packages. Model selection was done using AIC [18] corrected for small sample size (AICc) within the package AICcmodavg. To evaluate the viral shedding or load, Ct-values from the Matrix RRT-PCR runs that are proportional to the RNA copy numbers from original samples were used. First, we analyzed the variation in Ct-values in pre-challenged and naïve controls for the different treatment groups with different time periods between infections. The strategy was to use linear models and include individuals as random effect using mixed models (package GAMM) due to repeated sampling of the same individuals over the course of infection. The models that were evaluated included the factors: dpi, treatment group, both factors and the interaction. Ct-values from 1 dpi were not included in the analysis as they likely represented residual inoculum rather than true virus replication (at 1 dpi the Ct-values were close to 40). Models including the random effect of individual and the additive effect of dpi were tested but the increased complexity of the models was penalized based on the AICc and some of them had convergence problems. In the same way, the variation of Ct-values from all H3N8 control groups including age as factor was assessed. For the heterologous re-challenge, the variation in Ct-values from control and pre-challenged groups for each of the different viruses was evaluated as described before. The Area Under the Curve (AUC) was calculated by using the Ct-values after subtracting them from the cut-off value of 40 as previously done [22]. The total duration of infection or shedding was estimated by counting the days between inoculation and last positive virus isolations in CL swabs (which also includes cases of intermittent shedding). Birds that died or were euthanized before 14 dpi were not included in analysis. AUC and duration of infection between groups was compared using Krustal-Wallis test.

To study the correlation between AUC and duration of infection the Pearson correlation test was performed followed by a linear regression. To explore the relation between HA similarity and the degree of protection, we estimated the amino acid distance between the H3 and the different HA of re-challenges and the relative reduction in the duration of infection within groups. The amino acid distance was then correlated with the reduction in shedding per group where the mean and SE were estimated using a bootstrapping approach.

Next, to explore the influence of different variables on isolation success (binomial response: successful or unsuccessful) we used Generalized Linear Mixed Models (GLMM) as in [50]. The explanatory variables included in the models were: Ct-value, sample type (OP and CL swabs), dpi and treatment (pre-challenged or naïve individuals which means samples from a primary or secondary infection). Additionally, since individual birds were re-sampled and samples are not independent we added the individual as a random effect in the models. Interactions were not included to avoid convergence problems.

Last, MN antibody titers (as log_2_ transformed) at a single day of sampling were compared between groups using the Krustal-Wallis test and the paired t-test was used to compare values in two different days within groups. Samples with an MN titer lower than 20 were arbitrarily given a titer of log_2_ (2.5) for the model testing. The variation in antibody titers to H3 by MN was explored based on the different sampling occasions (dpi) and groups also using Generalized Additive Mixed Models in the package GAMM.

One duck died (12 dpi in group H6N2) and swabs were already IAV negative; necropsy showed no gross lesions caused by LPIAV. Another duck was euthanized due to difficulty walking which was associated with husbandry in captivity (6 dpi in group H3N8 X H6N8). These birds were not included in the analysis. The H3N8 x H12N5 group was not included in the analysis because of poor replication of this virus in the pre-exposed and naïve groups. All relevant data are available as Supporting Information files (Supplementary tables and S1 Dataset).

## Supporting information

S1 Fig Variation in the total shedding estimated by the Area under the curve (AUC) for the different treatment groups. Homologous challenge groups infected at different time intervals and infection in naïve control groups are shown in top panels. Heterologous challenge groups are in lower panels. Box plots indicate the distribution of the values where the horizontal lines inside the boxes show the median value.
(EPS)

S2 Fig Oropharyngeal shedding showing the variation in intensity of shedding (40—Ct-values), points indicate the mean per group and s.e. of the mean (SE) for the different groups. Homologous challenge groups infected at different time intervals are shown in top panels. Heterologous challenge groups are in lower panels. Continuous lines indicate the control groups and discontinuous lines indicate the H3N8 pre-challenged groups. * A single positive isolate from OP swab was an H10N7 detected at 0 dpi in the H10N7 control group. This duck was the last of a total of five to be inoculated in that isolator and this isolation was attributed to contamination with the inoculum. No birds had been exposed to H10N7 prior to this day.
(EPS)

S3 Fig Variation in H12N5 shedding (40—Ct-values), points indicate the mean per group and s.e. of the mean (SE) for the different groups Ct-values. Birds were pre-challenged (dashed lines) or not (solid lines) with H3N8 and challenged with H12N5. There was limited replication and shedding of the virus on the individuals inoculated with the H12N5. Only five CL swabs were positive by virus isolation while eight of the oropharyngeal swabs were positive by virus isolation. a) CL swabs and b) OP swabs.
(EPS)

S4 Fig Variation in Ct-values (primary y-axis) and Matrix RNA gene copies (secondary y-axis) over the course of infection for the H3N8 pre-challenge group. (EPS)

S1 Table Study design indicating the viruses used to inoculate each group, timeline and samples taken. NA: Not applicable; ✔ OP and CL swab sampling at 0–8 and 10, 12 and 14 dpi; * blood samples at 0 and 14 dpi; § additional blood sample taken.
(PDF)

S2 Table H3N8 x H3N8 homologous challenge– 5 week interval between infections. Model selection for the evaluation of variation in Ct-values in H3N8 pre-challenged groups compared to control groups of the same age challenged with the H3N8 virus strain. The terms included in each model are indicated with a “+” and “*” indicates the model that includes the terms and the interaction, “np” indicate the number of parameters. The best-ranked model, with lowest AICc, and significant p-values in the models are shown in bold. **A)** Model selection. **B)** Model showing the significance estimates.
(PDF)

S3 Table H3N8 x H3N8 homologous challenge– 11 week interval between infections. **A)** Model selection. **B)** Model showing the significance estimates.
(PDF)

S4 Table H3N8 x H3N8 homologous group– 15 week interval between infections. **A)** Model selection. **B)** Model showing the significance estimates.
(PDF)

S5 Table Mean total shedding (Area Under the Curve) and duration of infection with SE calculated for all challenge groups. (PDF)

S6 Table Models exploring the age effect on Ct-values after H3N8 infection. **A)** Model selection. **B)** Model showing the significance estimates for the different age classes. The terms included in each model are indicated with a “+” and “*” indicates the model that includes the terms and the interaction, “np” indicate the number of parameters. The best-ranked model, with lowest AICc, and significant p-values in the models are shown in bold.
(PDF)

S7 Table Heterologous challenge: H3N8 x H4N5 and H4N5 control groups. Model selection for the evaluation of variation in Ct-values in H3N8 pre-challenged groups compared to control groups challenged with the same virus strain. The terms included in each model are indicated with a “+” and “*” indicates the model that includes the terms and the interaction. “np” indicate the number of parameters. The best-ranked model, with lowest AICc, and significant p-values in the models are shown in bold. **A)** Model selection. **B)** Model showing the significance estimates.
(PDF)

S8 Table Heterologous challenge: H3N8 x H10N7 and H10N7 control groups. **A)** Model selection. **B)** Model showing the significance estimates.
(PDF)

S9 Table Heterologous challenge: H3N8 x H6N2 and H6N2 control groups. **A)** Model selection. **B)** Model showing the significance estimates.
(PDF)

S10 Table Models exploring the parameters influencing isolation success. **A)** Model in bold indicates the one with lowest AICc. The terms included in each model are indicated with a “+” and “*” indicates the model that includes the terms and the interaction, “np” indicate the number of parameters. Ct-val. stands for Ct-value, ST stands for sample type and Treat. stands for treatment whether it is primary or secondary infection. **B)** Model showing the significance estimates.
(PDF)

S11 Table Mean log_2_ transformed H3 titers and SE on groups pre-challenged with H3N8 at time point previous to re-infection (0 dpi) and at 14 dpi. (PDF)

S12 Table Models exploring the variation in H3 MN responses and titers. **A)** Model in bold indicates the one with lowest AICc. **B)** Model showing the significance estimates.
(PDF)

S1 Appendix Panel of antigens used for MN at the termination of the experiment for Fig 8. (PDF)

S1 Dataset Data files from the IAV experimental infections. Data on virus intensity (Ct-values) and isolation from the pre-challenge and the long-term homologous challenges (11 and 15 week intervals) is presented in separated different worksheets. Data on Ct-values and virus isolation for the heterologous challenges and the H3N8 x H3N8 (5- week interval) is presented in the same worksheet. Columns indicate: Sample ID, Band number (individual identifier for each duck), day post infection (Day.pi), Ct-values from the matrix RRT-PCR and isolation data for cloacal (CL) and oropharyngeal (OP) samples. NP-Elisa values are presented as the S/N ratio and the 1- S/N ratio was used for the graphs. NA denotes “not applicable”.
(XLSX)
