# Supplementary material for: Competition between influenza A virus subtypes through heterosubtypic immunity modulates re-infection and antibody dynamics in the mallard duck
Source: PLoS Pathog. 2017 Jun 22;13(6):e1006419. doi: 10.1371/journal.ppat.1006419 (PMC5481145; doi:10.1371/journal.ppat.1006419)
Supplement: S1 Table — NA: Not applicable; ✔ OP and CL swab sampling at 0–8 and 10, 12 and 14 dpi; * blood samples at 0 and 14 dpi; § additional blood sample taken. (PDF) [file ppat.1006419.s005.pdf]

Supporting Information:

Influenza A virus immunity and subtype competition in mallards

Neus Latorre-Margalef, Justin D. Brown, Alinde Fojtik, Rebecca L. Poulson, Deborah Carter, Monique Franca, David E. Stallknecht

DOI: 10.1371/journal.ppat.1006419

S1 Table.

	Challenges	I (4 weeks of age)	II (9 weeks of age)		III (15 weeks of age)	III (19 weeks of age)
**Group**	**N birds**	**0 dpi**	**35 dpi (5 weeks)**	**57 dpi**	**80 dpi (11 weeks)**	**108 dpi (15 weeks)**
H3N8 x H3N8 (5 week interval)	5	H3N8 * ✔	H3N8 * ✔	-	-	-
controls H3N8 (9 weeks of age)	5	*NA §*	H3N8 * ✔	-	-	-
H3N8 x H3N8 (11 week interval)	5	H3N8 * ✔	*NA §*	*NA §*	H3N8 * ✔	-
controls H3N8 (15 weeks of age)	5	*NA §*	*NA §*	*NA §*	H3N8 * ✔	-
H3N8 x H3N8 (15 week interval)	5	H3N8 * ✔	*NA §*	*NA §*	*NA §*	H3N8 * ✔
controls H3N8 (19 weeks of age)	5	*NA §*	*NA §*	*NA §*	*NA §*	H3N8 * ✔
H3N8 x H4N5	5	H3N8 * ✔	H4N5 * ✔	-	-	-
controls H4N5	5	*NA §*	H4N5 * ✔	-	-	-
H3N8 x H10N7	5	H3N8 * ✔	H10N7 * ✔	-	-	-
controls H10N7	5	*NA §*	H10N7 * ✔	-	-	-
H3N8 x H6N2	5 (4 final)	H3N8 * ✔	H6N2 * ✔	-	-	-
controls H6N2	5 (4 final)	*NA §*	H6N2 * ✔	-	-	-
H3N8 x H12N5	5	H3N8 * ✔	H12N5 * ✔	-	-	-
controls H12N5	5	*NA §*	H12N5 * ✔	-	-	-

NA: Not applicable

✔ OP and CL swab sampling at 0-8 and 10, 12 and 14 dpi

* blood samples at 0 and 14 dpi

§ additional blood sample taken
