# Supplementary material for: Competition between influenza A virus subtypes through heterosubtypic immunity modulates re-infection and antibody dynamics in the mallard duck
Source: PLoS Pathog. 2017 Jun 22;13(6):e1006419. doi: 10.1371/journal.ppat.1006419 (PMC5481145; doi:10.1371/journal.ppat.1006419)
Supplement: S2 Table — Model selection for the evaluation of variation in Ct-values in H3N8 pre-challenged groups compared to control groups of the same age challenged with the H3N8 virus strain. The terms included in each model are indicated with a “+” and “*” indicates the model that includes the terms and the interaction, “np” indicate the number of parameters. The best-ranked model, with lowest AICc, and significant p-values in the models are shown in bold. A) Model selection. B) Model showing the significance estimates. (PDF) [file ppat.1006419.s006.pdf]

Supporting Information:

Influenza A virus immunity and subtype competition in mallards

Neus Latorre-Margalef, Justin D. Brown, Alinde Fojtik, Rebecca L. Poulson, Deborah Carter, Monique Franca, David E. Stallknecht
DOI: 10.1371/journal.ppat.1006419

S2 Table.

A)

Models	*DPI*	*Group*	*DPI * Group*	*np*	*AICc*	*ΔAICc*	*AICc weights*
**1**	+	+	+	**6**	**289.26**	**0**	**0.843**
2	+	+		5	292.62	3.36	0.157
3	+			4	313.05	23.79	0.000
4		+		4	322.53	33.27	0.000

B)

	**Value**	**SE**	**DF**	**t-value**	**p-value**
Intercept Pre-exposed	37.68	3.03	40	12.41	**< 0.001**
Group control	-15.71	3.27	8	-4.79	**0.001**
Day PI	-0.02	0.52	40	-0.041	0.967
Group * Day PI	1.32	1.32	40	2.40	**0.020**
