# Supplementary material for: Competition between influenza A virus subtypes through heterosubtypic immunity modulates re-infection and antibody dynamics in the mallard duck
Source: PLoS Pathog. 2017 Jun 22;13(6):e1006419. doi: 10.1371/journal.ppat.1006419 (PMC5481145; doi:10.1371/journal.ppat.1006419)
Supplement: S4 Table — A) Model selection. B) Model showing the significance estimates. (PDF) [file ppat.1006419.s008.pdf]

Supporting Information:

Influenza A virus immunity and subtype competition in mallards

Neus Latorre-Margalef, Justin D. Brown, Alinde Fojtik, Rebecca L. Poulson, Deborah Carter, Monique Franca, David E. Stallknecht
DOI: 10.1371/journal.ppat.1006419

S4 Table.

A)

Models	*DPI*	*Group*	*DPI *Group*	*np*	*AICc*	*ΔAICc*	*AICc weights*
**1**	+	+	+	**6**	**213.93**	**0**	**0.446**
2	+			4	213.98	0.05	0.435
3	+	+		5	216.57	2.64	0.119
4		+		4	226.21	12.28	0.001

B)

	**Value**	**SE**	**DF**	**t-value**	**p-value**
Intercept Pre-exposed	45.72	7.63	25	5.98	**<0.001**
Group control	-17.97	7.79	6	-2.30	0.060
Day PI	-0.2.17	1.34	25	-1.61	0.119
Group * Day PI	3.16	1.36	25	2.31	**0.029**
