# Supplementary material for: Competition between influenza A virus subtypes through heterosubtypic immunity modulates re-infection and antibody dynamics in the mallard duck
Source: PLoS Pathog. 2017 Jun 22;13(6):e1006419. doi: 10.1371/journal.ppat.1006419 (PMC5481145; doi:10.1371/journal.ppat.1006419)
Supplement: S5 Table — (PDF) [file ppat.1006419.s009.pdf]

Supporting Information:

Influenza A virus immunity and subtype competition in mallards

Neus Latorre-Margalef, Justin D. Brown, Alinde Fojtik, Rebecca L. Poulson, Deborah Carter, Monique Franca, David E. Stallknecht
DOI: 10.1371/journal.ppat.1006419

S5 Table.

Group	Mean AUC	SE AUC	Mean Duration	SE Duration
H3N8 pre-challenge	72.74	5.22	8.4	0.87
H3N8 x H3N8 (5 weeks interval)	6.62	0.71	0	0
controls H3N8 (9 weeks of age)	101.19	6.99	8.4	1.6
H3N8 x H3N8 (11 weeks interval)	6.38	2.69	0	0
controls H3N8 (15 weeks of age)	53.05	7.1	6	0.89
H3N8 x H3N8 (15 weeks interval)	4.96	3.19	0	0
controls H3N8 (19 weeks of age)	45.25	4.64	4.8	0.49
H3N8 x H4N5	8.63	3.46	2.4	1.6
controls H4N5	64.21	9.3	8.8	1.33
H3N8 x H10N7	30.78	7.62	3.6	0.98
controls H10N7	66.57	2.49	7.6	1.33
H3N8 x H6N2 corrected	36.76	7.58	5	0.58
controls H6N2	86.5	2.58	7	1.29
