# Supplementary material for: Competition between influenza A virus subtypes through heterosubtypic immunity modulates re-infection and antibody dynamics in the mallard duck
Source: PLoS Pathog. 2017 Jun 22;13(6):e1006419. doi: 10.1371/journal.ppat.1006419 (PMC5481145; doi:10.1371/journal.ppat.1006419)
Supplement: S6 Table — A) Model selection. B) Model showing the significance estimates for the different age classes. The terms included in each model are indicated with a “+” and “*” indicates the model that includes the terms and the interaction, “np” indicate the number of parameters. The best-ranked model, with lowest AICc, and significant p-values in the models are shown in bold. (PDF) [file ppat.1006419.s010.pdf]

Supporting Information:

Influenza A virus immunity and subtype competition in mallards

Neus Latorre-Margalef, Justin D. Brown, Alinde Fojtik, Rebecca L. Poulson, Deborah Carter, Monique Franca, David E. Stallknecht
DOI: 10.1371/journal.ppat.1006419

S6 Table.

A)

Models	*DPI*	*Group*	*DPI *Group*	*np*	*AICc*	*ΔAICc*	*AICc weights*
**1**	+	+		**5**	**968.27**	**0**	**0.798**
2	+	+	+	6	971.02	2.75	0.202
3	+			4	983.54	15.27	0.000
4		+		4	1094.5	126.21	0.000

B)

	**Value**	**SE**	**DF**	**t-value**	**p-value**
Intercept (4 weeks of age)	24.21	0.67	150	35.84	**<0.001**
Group control (9 weeks)	-1.57	0.73	21	-2.15	**0.043**
Group control (15 weeks)	1.33	0.76	21	1.74	0.095
Group control (19 weeks)	2.37	0.79	21	2.99628	**0.006**
Day PI	1.19	0.08	150	13.55	**<0.001**
