# Supplementary material for: Competition between influenza A virus subtypes through heterosubtypic immunity modulates re-infection and antibody dynamics in the mallard duck
Source: PLoS Pathog. 2017 Jun 22;13(6):e1006419. doi: 10.1371/journal.ppat.1006419 (PMC5481145; doi:10.1371/journal.ppat.1006419)
Supplement: S7 Table — Model selection for the evaluation of variation in Ct-values in H3N8 pre-challenged groups compared to control groups challenged with the same virus strain. The terms included in each model are indicated with a “+” and “*” indicates the model that includes the terms and the interaction. “np” indicate the number of parameters. The best-ranked model, with lowest AICc, and significant p-values in the models are shown in bold. A) Model selection. B) Model showing the significance estimates. (PDF) [file ppat.1006419.s011.pdf]

Supporting Information:

Influenza A virus immunity and subtype competition in mallards

Neus Latorre-Margalef, Justin D. Brown, Alinde Fojtik, Rebecca L. Poulson, Deborah Carter, Monique Franca, David E. Stallknecht
DOI: 10.1371/journal.ppat.1006419

S7 Table.

A)

Models	*DPI*	*Group*	*DPI *Group*	*np*	*AICc*	*ΔAICc*	*AICc weights*
**1**	+	+	+	**6**	**258.17**	**0**	**0.603**
2	+	+		5	259.84	1.67	0.262
3	+			4	262.04	3.87	0.087
4		+		4	263.2	5.03	0.049

B)

	**Value**	**SE**	**DF**	**t-value**	**p-value**
Intercept Pre-exposed	38.93	3.24	34	11.98	**< 0.001**
Group control	-10.42	3.56	7	-2.92	**0.022**
Day PI	-0.73	0.64	34	-1.12	**< 0.001**
Group * Day PI	1.39	0.68	34	2.03	**0.049**
