# Supplementary material for: Competition between influenza A virus subtypes through heterosubtypic immunity modulates re-infection and antibody dynamics in the mallard duck
Source: PLoS Pathog. 2017 Jun 22;13(6):e1006419. doi: 10.1371/journal.ppat.1006419 (PMC5481145; doi:10.1371/journal.ppat.1006419)
Supplement: S8 Table — A) Model selection. B) Model showing the significance estimates. (PDF) [file ppat.1006419.s012.pdf]

Supporting Information:

Influenza A virus immunity and subtype competition in mallards

Neus Latorre-Margalef, Justin D. Brown, Alinde Fojtik, Rebecca L. Poulson, Deborah Carter, Monique Franca, David E. Stallknecht
DOI: 10.1371/journal.ppat.1006419

S8 Table.

A)

Models	*DPI*	*Group*	*DPI *Group*	*np*	*AICc*	*ΔAICc*	*AICc weights*
**1**	**+**	+		**5**	**332.44**	**0**	**0.745**
2	+	+	+	6	334.59	2.15	0.254
3	+			4	346.44	14	0.001
4		+		4	356.71	24.27	0.000

B)

	**Value**	**SE**	**DF**	**t-value**	**p-value**
Intercept Pre-exposed	29.27	1.21	47	24.06	**< 0.001**
Group control	-4.85	1.06	8	-4.57	**0.001**
Day PI	1.11	0.19	47	5.66	**< 0.001**
