# Supplementary material for: Competition between influenza A virus subtypes through heterosubtypic immunity modulates re-infection and antibody dynamics in the mallard duck
Source: PLoS Pathog. 2017 Jun 22;13(6):e1006419. doi: 10.1371/journal.ppat.1006419 (PMC5481145; doi:10.1371/journal.ppat.1006419)
Supplement: S9 Table — A) Model selection. B) Model showing the significance estimates. (PDF) [file ppat.1006419.s013.pdf]

Supporting Information:

Influenza A virus immunity and subtype competition in mallards

Neus Latorre-Margalef, Justin D. Brown, Alinde Fojtik, Rebecca L. Poulson, Deborah Carter, Monique Franca, David E. Stallknecht
DOI: 10.1371/journal.ppat.1006419

S9 Table.

A)

Models	*DPI*	*Group*	*DPI *Group*	*np*	*AICc*	*ΔAICc*	*AICc weights*
**1**	**+**	**+**		**5**	**492.61**	**0**	**0.595**
2	+	+	+	6	493.86	1.25	0.318
3	+			4	496.46	3.85	0.087
4		+		4	560.91	68.3	0.000

B)

	**Value**	**SE**	**DF**	**t-value**	**p-value**
Intercept Pre-exposed	24.85	0.94	76	26.20	**< 0.001**
Group control	-2.52	0.91	8	-2.75	**0.024**
Day PI	1.37	0.13	76	10.36	**< 0.001**
