# Supplementary material for: Competition between influenza A virus subtypes through heterosubtypic immunity modulates re-infection and antibody dynamics in the mallard duck
Source: PLoS Pathog. 2017 Jun 22;13(6):e1006419. doi: 10.1371/journal.ppat.1006419 (PMC5481145; doi:10.1371/journal.ppat.1006419)
Supplement: S10 Table — A) Model in bold indicates the one with lowest AICc. The terms included in each model are indicated with a “+” and “*” indicates the model that includes the terms and the interaction, “np” indicate the number of parameters. Ct-val. stands for Ct-value, ST stands for sample type and Treat. stands for treatment whether it is primary or secondary infection. B) Model showing the significance estimates. (PDF) [file ppat.1006419.s014.pdf]

Supporting Information:

Influenza A virus immunity and subtype competition in mallards

Neus Latorre-Margalef, Justin D. Brown, Alinde Fojtik, Rebecca L. Poulson, Deborah Carter, Monique Franca, David E. Stallknecht
DOI: 10.1371/journal.ppat.1006419

S10 Table.

A)

Models	*Ct-val.*	*ST*	*Treat.*	*DPI*	*np*	*AICc*	*ΔAICc*	*AICc weights*
**1**	**+**		**+**	**+**	**5**	**216.87**	**0.000**	**0.626**
2	+	+	+	+	6	217.93	1.060	0.369
3	+			+	4	226.42	9.550	0.005
4	+		+		4	237.4	20.530	0.000
5	+	+			5	239.44	22.570	0.000
6	+				3	238.64	21.770	0.000
7	+	+	+		4	240.71	23.840	0.000
8		+		+	4	285.6	68.730	0.000
9				+	3	287.92	71.050	0.000
10			+		3	389.94	173.070	0.000
11		+	+		4	391.23	174.360	0.000
12					2	399.73	182.860	0.000
13		+			3	401.28	184.410	0.000

B)

	**Value**	**SE**	**z-value**	**p-value**
Intercept (primary infection)	17.28	0.29	59.2	**< 0.001**
DPI	-0.32	0.005	-63.5	**< 0.001**
Ct-value	-0.41	0.005	-76.4	**< 0.001**
Secondary infection	-1.85	0.005	-365.5	**< 0.001**
