# Supplementary material for: Competition between influenza A virus subtypes through heterosubtypic immunity modulates re-infection and antibody dynamics in the mallard duck
Source: PLoS Pathog. 2017 Jun 22;13(6):e1006419. doi: 10.1371/journal.ppat.1006419 (PMC5481145; doi:10.1371/journal.ppat.1006419)
Supplement: S11 Table — (PDF) [file ppat.1006419.s015.pdf]

Supporting Information:

Influenza A virus immunity and subtype competition in mallards

Neus Latorre-Margalef, Justin D. Brown, Alinde Fojtik, Rebecca L. Poulson, Deborah Carter, Monique Franca, David E. Stallknecht

DOI: 10.1371/journal.ppat.1006419

S11 Table.

	H3xH3 (5w)	H3xH4	H3xH10	H3xH6	H3xH3 (11w)	H3xH3 (15w)
**Day 0 pi**	5.82 ± 0.85	6.32 ± 0-63	4.57 ± 0.22	6.30 ± 0.5	6.12 ± 0.66	6.32 ± 0.31
**Day 14 pi**	5.92 ± 0.5	7.32 ± 0.63	5.66 ± 0.51	5.90 ± 0.28	8.50 ± 0.58	7.70 ± 0.36
