# Supplementary material for: Competition between influenza A virus subtypes through heterosubtypic immunity modulates re-infection and antibody dynamics in the mallard duck
Source: PLoS Pathog. 2017 Jun 22;13(6):e1006419. doi: 10.1371/journal.ppat.1006419 (PMC5481145; doi:10.1371/journal.ppat.1006419)
Supplement: S12 Table — A) Model in bold indicates the one with lowest AICc. B) Model showing the significance estimates. (PDF) [file ppat.1006419.s016.pdf]

Supporting Information:

Influenza A virus immunity and subtype competition in mallards

Neus Latorre-Margalef, Justin D. Brown, Alinde Fojtik, Rebecca L. Poulson, Deborah Carter, Monique Franca, David E. Stallknecht
DOI: 10.1371/journal.ppat.1006419

S12 Table.

A)

Models	*DPI*	*Group*	*DPI *Group*	*np*	*AICc*	*ΔAICc*	*AICc weights*
**1**	**+**			**4**	**166.83**	**0**	**0.379**
2	+	+		7	167.01	0.18	0.347
3	+	+	+	10	168.59	1.76	0.157
4		+		6	169.19	2.36	0.117

B)

	**Value**	**SE**	**DF**	**t-value**	**p-value**
Intercept Pre-exposed	5.09	0.37	30	14.09	**< 0.001**
Day pi 35	0.02	0.009	30	2.31	**0.02**
