# Supplementary material for: Competition between influenza A virus subtypes through heterosubtypic immunity modulates re-infection and antibody dynamics in the mallard duck
Source: PLoS Pathog. 2017 Jun 22;13(6):e1006419. doi: 10.1371/journal.ppat.1006419 (PMC5481145; doi:10.1371/journal.ppat.1006419)
Supplement: S1 Appendix — (PDF) [file ppat.1006419.s017.pdf]

Supporting Information:

Influenza A virus immunity and subtype competition in mallards

Neus Latorre-Margalef, Justin D. Brown, Alinde Fojtik, Rebecca L. Poulson, Deborah Carter, Monique Franca, David E. Stallknecht
DOI: 10.1371/journal.ppat.1006419

S1 Appendix.

A/mallard/MN/AI0-4823/2010 (H1N1)

A/mallard/MN/AI08-2755/2008 (H2N3)

A/mallard/MN/AI0-2593/2010 (H3N8)

A/mallard/MN/AI10-3208/2010 (H4N6)

A/mallard/MN/AI11-3933/2011 (H5N1)

A/mallard/MN/AI08-2721/2008 (H6N1)

A/mallard/MN/AI08-3770/2009 (H7N9)

A/mallard/MN/SG-01048/2008 (H8N4)

A/ruddy turnstone/DE/AI11-809/2011 (H9N2)

A/mallard/MN/SG-00999/2008 (H10N7)

A/mallard/MN/SG-00930/2008 (H11N9)

A/mallard/MN/SG-3285/2007 (H12N5)

A/blue-winged teal/TX/AI13-1028/2010 (H14N5)

A/wedge-tailed shearwater/Western Australia/2327/1983 (H15N6)
